# Synthesis and self-assembly of curcumin-modified amphiphilic polymeric micelles with antibacterial activity

**DOI:** 10.1186/s12951-021-00851-2

**Published:** 2021-04-13

**Authors:** Caio H. N. Barros, Dishon W. Hiebner, Stephanie Fulaz, Stefania Vitale, Laura Quinn, Eoin Casey

**Affiliations:** 1 School of Chemical and Bioprocess Engineering, University College Dublin, Dublin, Ireland; 2 Present Address: National Institute for Bioprocessing Research and Training (NIBRT), Dublin, Ireland; 3 Present Address: School of Pharmacy and Biomolecular Sciences, Irish Centre for Vascular Biology, Royal College of Surgeons in Ireland, Dublin, Ireland; 4 Present Address: Université de Strasbourg, CNRS, ISIS, 8 allée Gaspard Monge, 67000 Strasbourg, France

**Keywords:** Micelle, Curcumin, Biofilm, *Pseudomonas*

## Abstract

**Background:**

The ubiquitous nature of bacterial biofilms combined with the enhanced resistance towards antimicrobials has led to the development of an increasing number of strategies for biofilm eradication. Such strategies must take into account the existence of extracellular polymeric substances, which obstruct the diffusion of antibiofilm agents and assists in the maintenance of a well-defended microbial community. Within this context, nanoparticles have been studied for their drug delivery efficacy and easily customised surface. Nevertheless, there usually is a requirement for nanocarriers to be used in association with an antimicrobial agent; the intrinsically antimicrobial nanoparticles are most often made of metals or metal oxides, which is not ideal from ecological and biomedical perspectives. Based on this, the use of polymeric micelles as nanocarriers is appealing as they can be easily prepared using biodegradable organic materials.

**Results:**

In the present work, micelles comprised of poly(lactic-*co*-glycolic acid) and dextran are prepared and then functionalised with curcumin. The effect of the functionalisation in the micelle’s physical properties was elucidated, and the antibacterial and antibiofilm activities were assessed for the prepared polymeric nanoparticles against *Pseudomonas* spp*.* cells and biofilms. It was found that the nanoparticles have good penetration into the biofilms, which resulted in enhanced antibacterial activity of the conjugated micelles when compared to free curcumin. Furthermore, the curcumin-functionalised micelles were efficient at disrupting mature biofilms and demonstrated antibacterial activity towards biofilm-embedded cells.

**Conclusion:**

Curcumin-functionalised poly(lactic-*co*-glycolic acid)-dextran micelles are novel nanostructures with an intrinsic antibacterial activity tested against two *Pseudomonas* spp. strains that have the potential to be further exploited to deliver a secondary bioactive molecule within its core.

**Graphic Abstract:**

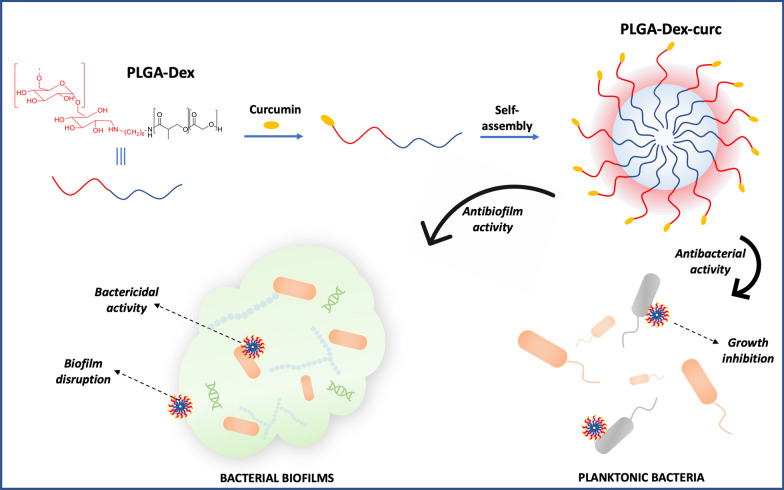

**Supplementary Information:**

The online version contains supplementary material available at 10.1186/s12951-021-00851-2.

## Background

Biofilms are communities of microorganisms physically encased within a matrix rich in biomolecules, commonly known as extracellular polymeric substances (EPS) [1]. The prevalence of biofilms in both the industrial and clinical settings is a constant source of concern given their harmful character. Besides the enhanced levels of host-threatening virulence factors produced by bacterial cells possessing the biofilm phenotype, the matrix’s physical protection further amplifies the resistance of biofilms [2, 3]. The thick gelatinous layer of biomolecules which encases the bacteria represents a significant hurdle for antibiotics; the penetration of foreign agents is in most part hindered by the dense network of secreted proteins, nucleic acids and polysaccharides that constitute the EPS [3].

Nanoparticles (NPs) are currently being employed as probes and drug nanocarriers with better performance at penetrating the biofilm matrix [4–6]; the possibility of choosing the chemical composition, surface topography and size of NPs widens the chances of achieving effective antibiofilm strategies [7]. Nevertheless, the use of NPs containing metals such as zinc, copper, silver, gold and iron has some drawbacks regarding their environmental fate and biocompatibility [8, 9]. Within this context, the synthesis of polymeric NPs gains a clear advantage, seeing that their organic nature allied with biodegradability would solve the aforementioned issues [10]. More specifically, polymeric micelles comprised of amphiphilic copolymers are especially interesting given the easiness of the preparation process, low cost and highly tuneable nature [11].

The preparation of amphiphilic copolymers and their self-assembly into micelles has many biological applications due to these systems’ capacity to encapsulate and deliver bioactive molecules [12, 13]. In the biofilm context, polymeric micelles can be quite useful for the delivery of antibiofilm agents. The use of polymers that provide stealth properties and prevent non-specific binding between surface groups and EPS components leads to enhanced biofilm penetration, thus improving the effectiveness of encapsulated water-insoluble compounds [14, 15]. Furthermore, the process of delivery itself can be designed to occur only when triggered by external stimuli. For example, pH- [16], enzyme- [17] and light-responsive [18] micelles are suitable for antibiofilm applications as specific conditions in the interior of the biofilm may trigger the disassembly of the micelle and the subsequent release of antimicrobials. On top of these advantages, the fact that many micelles can be prepared from biodegradable polymers is highly favourable for their use. Environmentally-friendly materials may increase the stability and biocompatibility of antibiotics, thereby protecting the bioactive compound from degradation once exposed to a biological system [10]. Synthetic polymers, such as polycaprolactone, and natural ones, such as chitosan, have been successfully used for the preparation of micelles that encapsulate antibiofilm agents [15, 19].

Although the encapsulation of antibiotics within the micellar hydrophobic core is a common approach for antimicrobial delivery, it may present some drawbacks. This includes the non-specific release or leakage of the loaded drug, especially when the compound has a somewhat hydrophilic character [10]. Moreover, the polymeric nanoparticle itself usually acts solely as a nanocarrier, not contributing to the antibacterial action in any way. This may represent a wasted potential for additional antibacterial action that might arise from the polymeric shell itself. To overcome these limitations and seize the full potential of polymeric nanocarriers, the bioactive compound (antimicrobial and/or antibiotic) can be covalently bonded to the polymeric structure itself, thus rendering it intrinsic activity against bacteria. The antibiotic and antimicrobial molecules can be linked to the polymeric chain's backbone as bioactive pendant groups that confer an overall bactericidal activity [20, 21]. Another methodology involves decorating the micelle shell with the bioactive compound, with the preparation of surfaces functionalised with an amphiphilic polymeric micelle which is further decorated with an antimicrobial peptide. These approaches do not covalently modify the polymer structure’s backbone, but rather add the antimicrobial units to the outer portion of the micelle [22]. In some cases, when the polymeric backbone (or side groups) are positively charged, no structural modification is necessary to induce the bactericidal effect. Chitosan-modified poly(d, l-lactide-*co*-glycolide) (Chitosan-PLGA) and Soluplus^®^ micelles, for instance, display inherent antibacterial and antibiofilm activities due to the positively charged chitosan, which causes an electrostatic imbalance to the negatively charged bacterial cell wall [23]. Another example of the charge-induced bactericidal effect is the quaternary ammonium salt-based micelles, which are intrinsically antimicrobial even below the critical micelle concentration (CMC), and act by decomposing the biofilm matrix and killing both planktonic and sessile cells [14].

With the aim of fabricating inherently antimicrobial micelles, the incorporation of antimicrobials into micelles’ polymeric structure is also a valid strategy. For example, triclosan and biguanide groups have been successfully incorporated into the polymeric backbone of NPs and have thereby conferred antibacterial activity [21]. The choice of the amphiphilic copolymer itself is also crucial for the success of an antimicrobial micelle; parameters such as partition coefficient (a parameter that describes the ratio of the concentration of a solute in two immiscible solvents) of each polymer, presence of reactive groups, biodegradability and interactions with the biofilm EPS must be considered.

For this study, poly(d, l-lactide-*co*-glycolide) (PLGA) was chosen as the hydrophobic moiety of the copolymer due to its good biocompatibility, low cost and ease of chemical modification [24]. Dextran, a naturally branched glucan, was selected as the micelle's hydrophilic shell due to its biodegradability and its easily functionalised chemical groups [25]. Dextran is a polysaccharide-rich in α(1 → 6) glucose links with high variability of linkages and branches. Its abundance of hydroxyl groups and the presence of a reducing end facilitates chemical modification of its structure, such as through the formation of esters, ethers, thiols, phosphates, among others [26]. Moreover, dextran is known for being compatible with the EPS polysaccharides [27], favouring increased micelle penetration into the EPS matrix. Covalent bonding of dextran with PLGA can thus generate a copolymer of amphiphilic nature that is able to naturally self-assemble into micelles.

In this paper, the main objective was to synthesise and characterise biodegradable antimicrobial micelles constituted of a PLGA-dextran copolymer featuring covalently linked curcumin, the active ingredient of *Curcuma* *longa* [28]. Curcumin is a phytochemical known for displaying a wide span of biological activities, including anti-cancer [29], anti-inflammatory [30] and outstanding antibacterial activities [31]. It has phenolic groups that can be used for derivatisation and is hydrophobic, making its administration as a free compound considerably hindered due to its low solubility and bioavailability [32]. Curcumin-functionalised PLGA-dextran micelles (PLGA-Dex_10_-curc) had their antibacterial and antibiofilm activities assessed against the model biofilm-forming strains *Pseudomonas* *putida* (*P.* *putida*) and *Pseudomonas* *fluorescens* (*P.* *fluorescens*) with the aim of introducing an environmentally friendly and biodegradable polymeric micelle with intrinsic bioactive properties.

## Results and discussion

### Polymeric micelles synthesis and characterisation

**Fig. 1:**
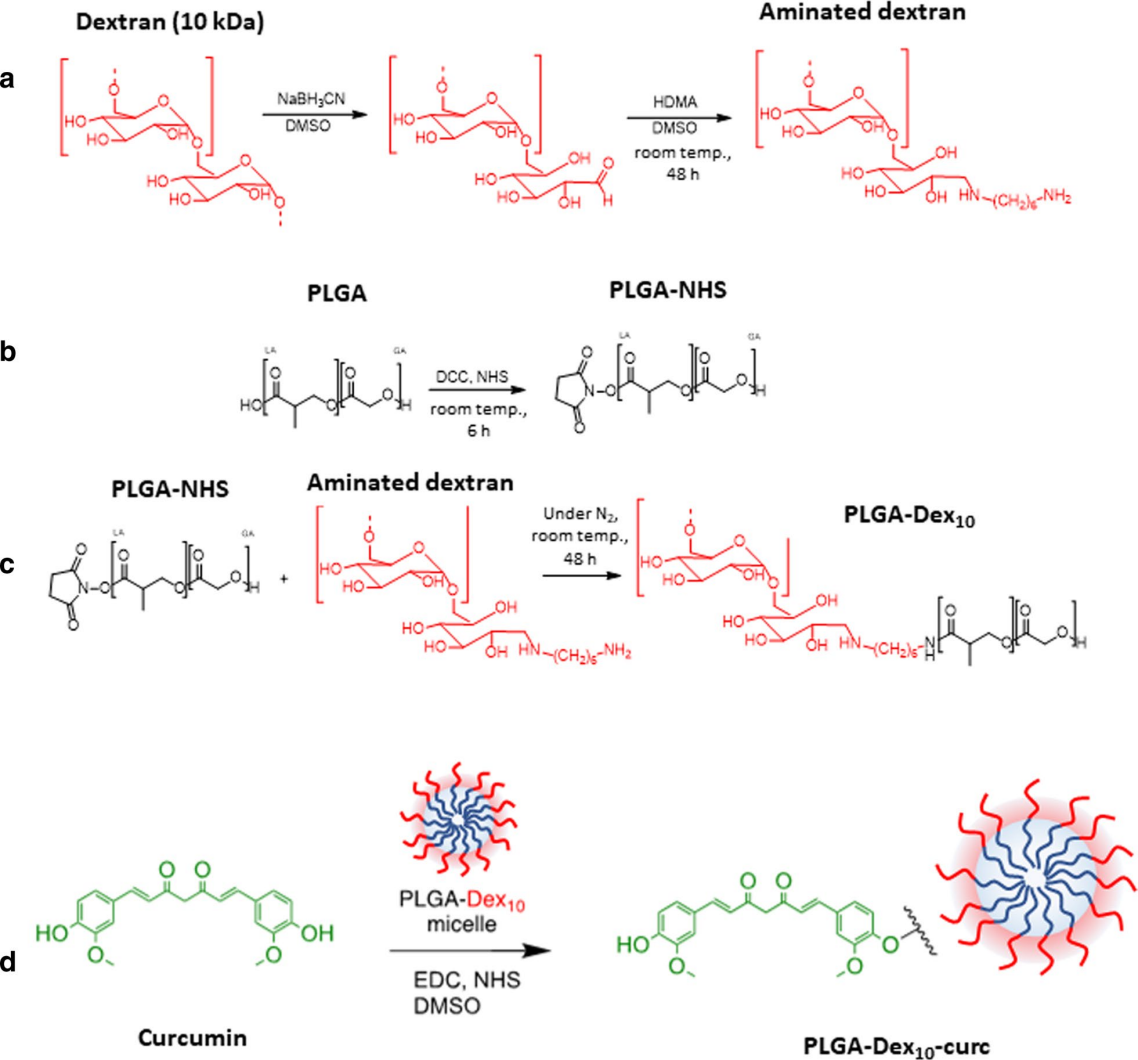
Reductive amination of dextran (**a**), activation of PLGA (**b**), preparation of copolymer PLGA-Dex_10_ (**c**) and preparation of PLGA-Dex_10_-curc (**d**)

The synthesis of the poly(d, l-lactide-*co*-glycolide)-dextran (PLGA-Dex_10_) copolymer requires activation of both dextran and PLGA prior to reaction. Dextran was aminated via reductive amination using sodium cyanoborohydrate (NaBH_3_CN) and hexamethylenediamine (HDMA), while poly(d, l-lactide-*co*-glycolide) with a carboxylic acid termination (PLGA-COOH) was activated with *N*-hydroxysuccinimide (NHS) (Fig. 1). The block copolymer can be synthesised by merely mixing both activated polymers in dimethyl sulfoxide (DMSO). The synthesised block copolymer is constituted of a hydrophobic moiety (PLGA), and a hydrophilic one (dextran) and its self-assembly in the form of micelles occurs spontaneously during the dialysis procedure, in which the DMSO from the reaction medium is slowly substituted by water. Additional file 1: Figure S1 shows the Fourier-transform infrared (FTIR) spectra of dextran, PLGA-COOH and the conjugated PLGA-Dex_10_ copolymer. The appearance of a C=O stretching vibrational mode at 1670 cm^−1^ confirms the presence of PLGA in the copolymer structure, whereas the broad O–H band at 3331 cm^−1^ is indicative of the presence of the hydroxyl groups from dextran. Bands in the region between 1600 cm^−1^ and 1000 cm^−1^ in the spectrum of PLGA-COOH had their intensities decreased in the spectrum of PLGA-Dex_10_ most probably due to a concentration effect, since dextran accounts for a significant part of the total mass of the copolymer.

**Fig. 2:**
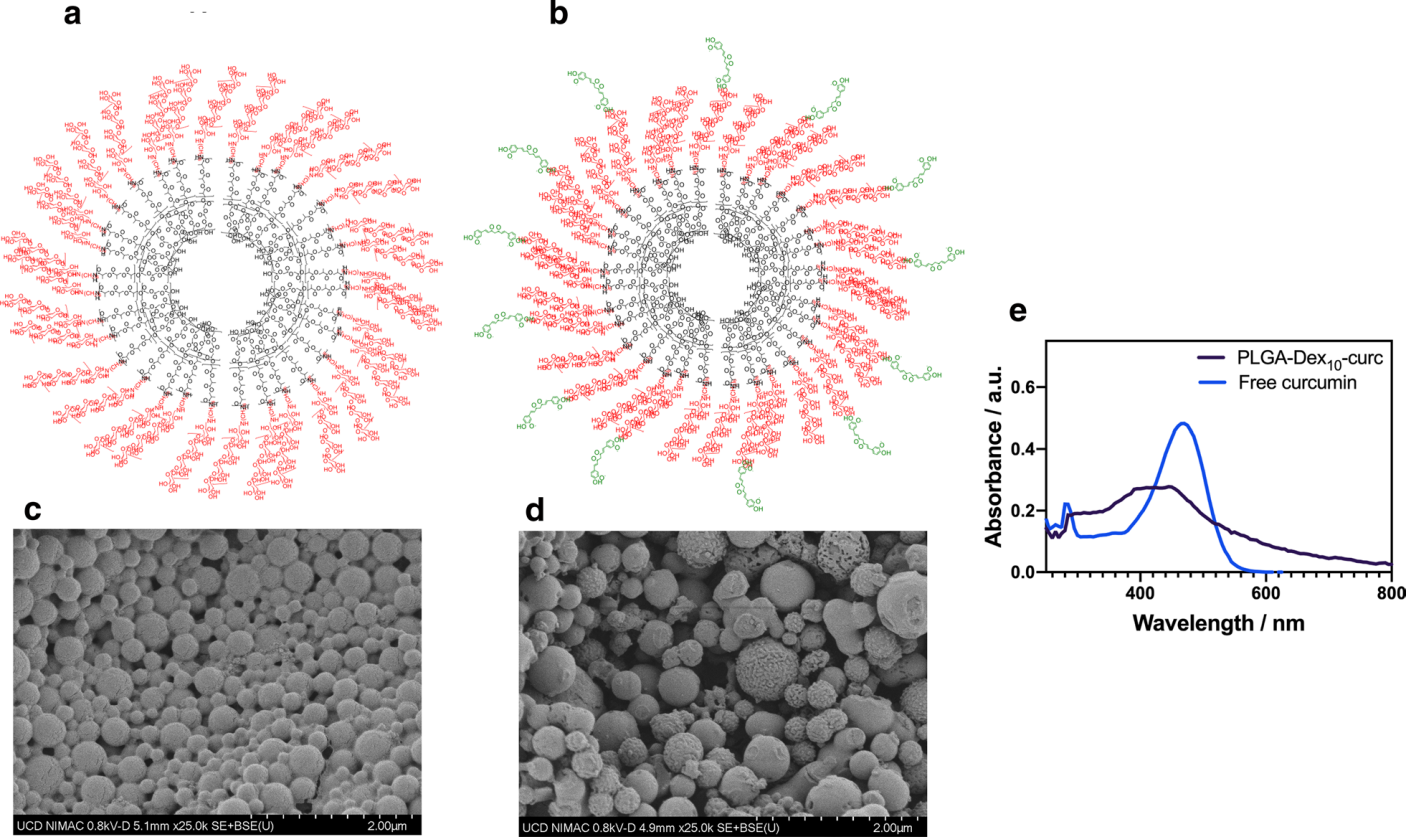
Schematic diagram and scanning electron microscopy images of PLGA-Dex_10_ (**a**, **c**), PLGA-Dex_10_-curc (**b**, **d**) and UV–Vis spectra of PLGA-Dex_10_-curc and free curcumin in water (**e**)

The self-assembled PLGA-Dex_10_ micelle has a hydrodynamic size of 113.0 ± 34.0 nm and zeta potential of − 6.0 ± 0.6 mV (Table 1). Despite the weak electrostatic stabilisation, the dispersions were stable for months, most likely due to steric stabilisation. Micrographs of this polymeric nanoparticle show much larger spheres than what could be predicted from dynamic light scattering (DLS) measurements, most probably due to the Ostwald ripening phenomenon that happens during the evaporation of the solvent onto the copper tape for scanning electron microscopy (SEM) analysis. This effect is characterised by small particles’ coalescence into larger particles, thus shifting the average size of the colloidal suspension toward higher values [33, 34]. Ostwald ripening is easily seen in all micrographs of Fig. 2, in which larger particles seem to be increasing in size at the expense of smaller ones.

**Table 1:** Hydrodynamic size and zeta potential of polymeric micelles

Sample	Size (DLS)/nm	Polydispersity	Zeta potential (mV)
PLGA-Dex_10_	113.0 ± 34.0	0.072	− 6.0 ± 0.6
PLGA-Dex_10_-curc	498.7 ± 35.4	0.162	+ 25.6 ± 0.8

The dextran shell of the micelle was then functionalised with curcumin to accentuate its antibacterial and antibiofilm activity. In contrast with more conventional approaches in which polymeric nanoparticles serve as a mere nanocarrier of encapsulated antimicrobials, here the intent is to produce nanocarriers which are intrinsically antimicrobial. The functionalisation using curcumin (PLGA-Dex_10_-curc) resulted in much larger hydrodynamic diameters (498.7 ± 35.4 nm). In addition to this, the micelles also seem to have a rougher surface, as shown in the micrograph in Fig. 2d. Surprisingly, these micelles became positively charged, attributed to the effect of residual *N*-(3-dimethylaminopropyl)-*N*′-ethylcarbodiimide hydrochloride (EDC), which is used in excess during the reaction and may have been adsorbed onto the nanoparticles. More prolonged dialysis procedures and exhaustive centrifugal washing could not shift the zeta potential from positive towards more negative values.

In order to confirm the success of the functionalisation with curcumin along with the purpose of estimating the antimicrobial loading into the micelles, the UV–Vis spectrum was acquired of PLGA-Dex_10_-curc using a PLGA-Dex_10_ micelle suspension as a blank. PLGA-Dex_10_-curc shows an absorption band at 425 nm arising from curcumin (Fig. 2e). Calculations of curcumin loading using standard calibration curves resulted in 1.8 ± 0.2% (w/w) curcumin loading for PLGA-Dex_10_-curc. Characterisation using FTIR revealed essential information to confirm the functionalisation steps further. As seen in Additional file 1: Figure S2 and Table S1, the insertion of curcumin into the dextran shell resulted in the appearance of a phenolic C–O band at 1179 cm^−1^, a C=C vibrational mode at 1629 cm^−1^ and an enolic C–O band at 1422 cm^−1^. The emergence of absorption bands belonging to key functional groups of curcumin molecules further confirms the success of the functionalisation reactions.

**Fig. 3:**
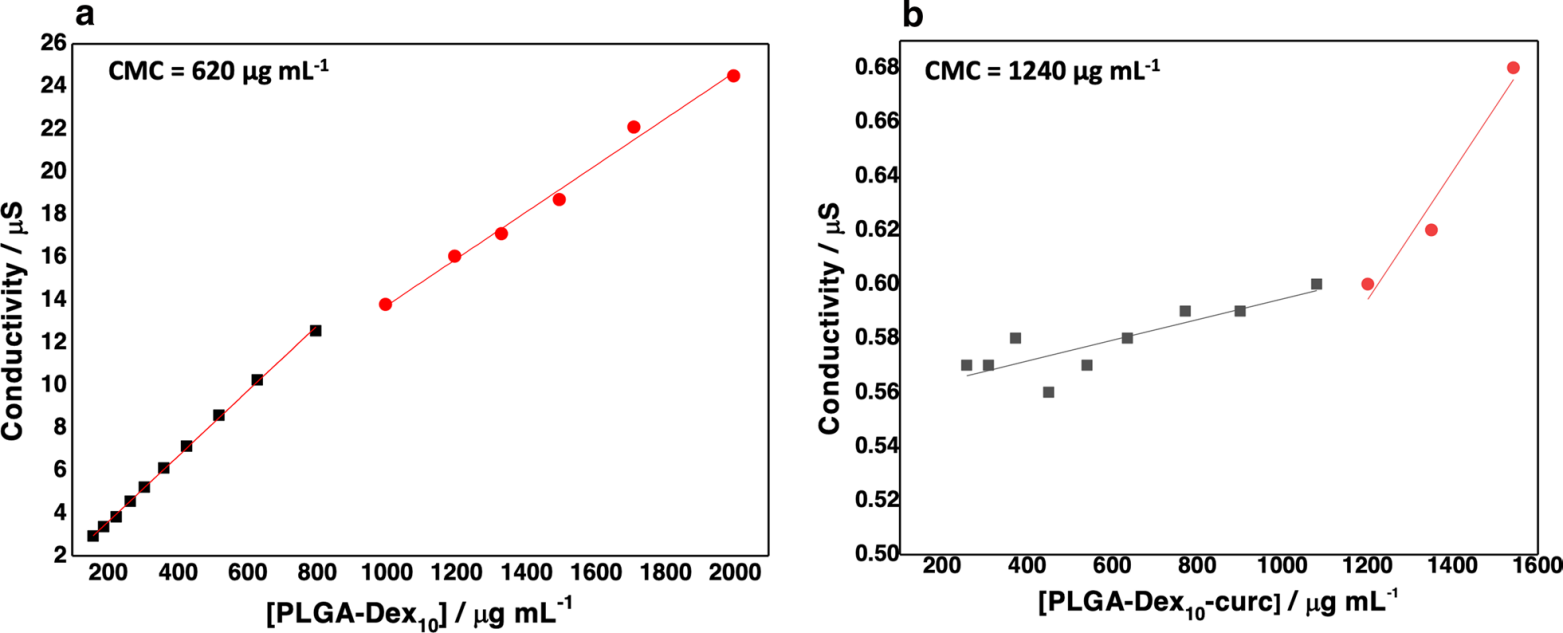
Estimation of critical micelle concentration of PLGA-Dex_10_ (**a**) and PLGA-Dex_10_-curc (**b**) using conductivity measurements in distilled water

An essential parameter for micelles’ characterisation is the CMC, which is the threshold concentration at which the individual molecules self-organise in a micellar structure [35]. Here, conductivity measurements of the copolymer solutions were utilised in order to determine the CMC of each micelle type. Increasing concentrations of the solute are expected to increase the conductivity of the solution linearly up to the CMC when subsequent addition of solute induces an attenuated increase in conductivity. Therefore, two linear trends are seen in Fig. 3, and the intersection of both is an estimation of the CMC. As seen in Fig. 3, both PLGA-Dex_10_ follows the expected trend. However, PLGA-Dex_10_-curc shows an even higher slope after the formation of micelles. This effect could be related to the hydrophobicity of curcumin, which lowers the system's conductivity as a whole. The estimated CMC values were 620 µg mL^−1^ and 1240 µg mL^−1^, for PLGA-Dex_10_ and PLGA-Dex_10_-curc, respectively. The hydrophobicity of curcumin contributed for the shift to higher CMC, as the difference of hydrophilicity between the PLGA core and the dextran shell was lessened. Additionally, the dextran shell functionalised with curcumin is larger and could mean that more units of the copolymer are needed to form a structured self-assembled micelle. The CMC and shape of amphiphilic polymeric micelles are intrinsically related to the length of each block polymer and the difference of the partition coefficient of these polymers [36, 37].

### Antibacterial and antibiofilm assays

**Fig. 4:**
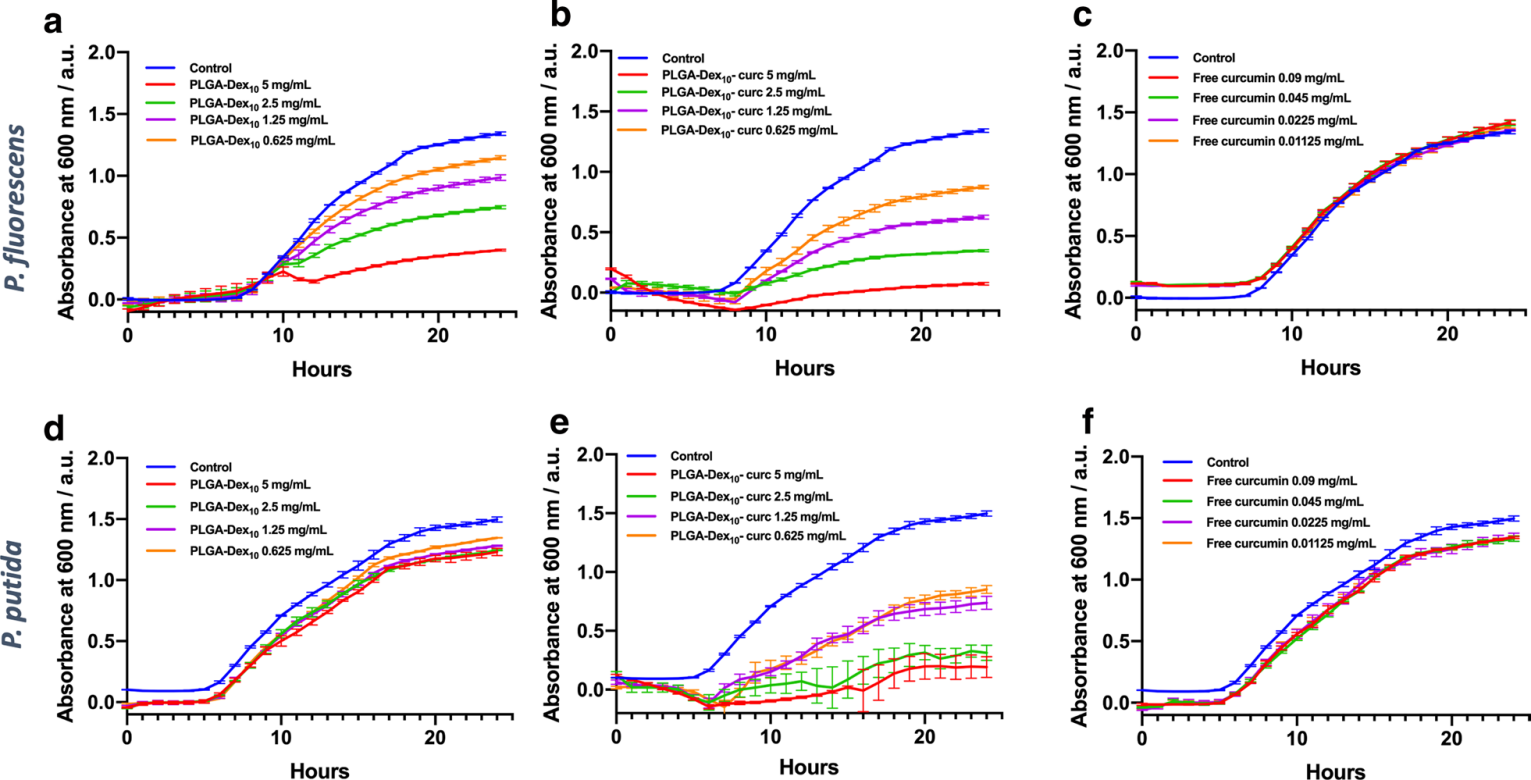
Bacterial growth kinetics of *P.* *fluorescens* (**a**–**c**) and *P.* *putida* (**d**–**f**) planktonic cells in the presence of micelles, curcumin-functionalised micelles and free curcumin. Error bars are expressed in terms of the standard error of the mean

In order to investigate the antibacterial effects of the two types of micelles synthesised, biofilm-forming *P.* *fluorescens* and *P.* *putida* strains were cultivated in 96-well plates in the presence of the test micelles at four different concentration values (0.62, 1.25, 2.50, 5.00 mg mL^−1^) for 24 h. Absorbance readings at 600 nm were taken every hour to assess bacterial growth (Fig. 4). As a means of comparison, free curcumin was also used at equivalent concentration values as found in the micelles.

The first noticeable result obtained from these experiments is that the PLGA-Dex_10_ micelle has an antimicrobial effect on its own against *P.* *fluorescens* (Fig. 4a), which is not seen for *P.* *putida* (Fig. 4d), likely due to inherent differences in either the phenotypic or genotypic features between each bacterial strain. Despite the well-known dextran biocompatibility, some papers have reported the antibacterial and antibiofilm effect of oligosaccharides towards some *Pseudomonas* strains [38, 39]. Here, it seems that the dextran shell slows down the growth rate of planktonic cells of *P.* *fluorescens*, not necessarily meaning that there is bacterial eradication. Conversely to the other growth inhibition patterns seen in Fig. 4, in this case, the growth rate is the same as in control conditions up to 10 h. After this time point, inhibition seems to be triggered. The insertion of curcumin on the dextran shell further intensified the antibacterial effect towards *P.* *fluorescens*; as well as conferred inhibition activity towards *P.* *putida*, which is noted by the concentration-dependent delay of bacterial growth (Fig. 4d–f).

Interestingly, free curcumin at equivalent concentrations was not able to inhibit bacterial growth to any extent. This fact is in line with what was already observed by our research group with the functionalisation of curcumin onto silica NPs [40]. This result suggests that the conjugation of curcumin on the micelle structure might have improved the natural product’s solubility and transport in the bacterial suspension, which in turn accentuated its antimicrobial capacity. The outstanding bacterial growth inhibition of PLGA-Dex_10_-curc can also be explained by its positive charge; it is known that positively charged nanoparticles are often very aggressive towards Gram-negative bacteria due to the interaction of the positively-charged nanoparticle with the negatively-charged bacterial cell wall [41].

The bactericidal effects induced by curcumin have been thoroughly studied, and the mechanisms through which they take place are plural. Membrane permeabilisation studies combined with electron microscopy point out that curcumin induces membrane damage and leakage of intracellular contents in Gram-positive and Gram-negative bacteria [42]. Other studies focused on transcriptomics suggest that curcumin also changes gene expression. For instance, the enzymes catalase and superoxide dismutase production is suppressed in *Dictyostelium* *discoideum*, which elevates the levels of reactive oxygen species (ROS) in the cell, affecting the life cycle and cell proliferation [43]. Proteins linked to virulence and pathogenicity such as elastases and proteases were also shown to be downregulated in *Pseudomonas* *aeruginosa* (*P.* *aeruginosa*) exposed to curcumin [44]. Additionally, molecules involved in the quorum sensing (QS) signalling process such as pyocyanin and *N*-acyl-homoserine lactones (AHLs) were also downregulated, which shows an extended effect on the biofilm-formation signalling pathways [44].

**Fig. 5:**
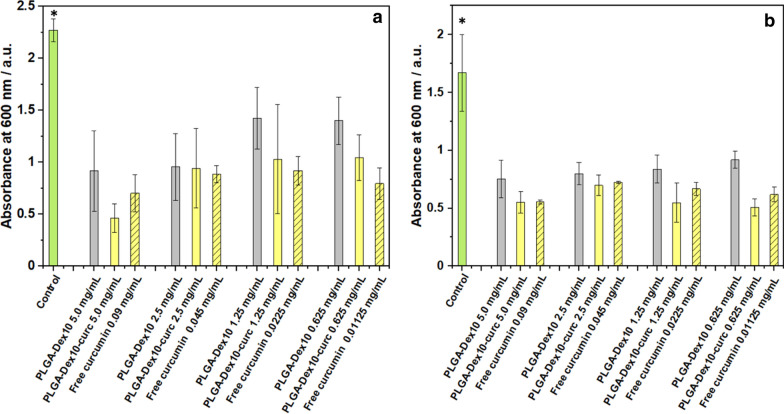
Biofilm biomass quantification using crystal violet staining after 24 h growth of Gram-negative *P.* *fluorescens* (**a**) and Gram-negative *P.* *putida* (**b**) in the presence of micelles, curcumin-functionalised micelles and free curcumin. Control corresponds to the addition of a bacterial culture medium instead of micelles/curcumin solution. Error bars are expressed in terms of standard deviation*.* Asterisk means statistical significance in comparison with all other conditions (p < 0.05)

Whereas the assessment of antibacterial activity has its importance, it is known that pathogenicity and drug resistance are intimately related to the biofilm-forming capabilities of bacterial strains [45]. Therefore, we then investigated the biofilm-inhibition capacity of PLGA-Dex_10_ and PLGA-Dex_10_-curc micelles. The anti-adhesion properties were first evaluated by estimating the biofilm biomass generated after 24 h of cultivation. Crystal violet staining was used to quantify the biomass formed in the presence of the micelles and free curcumin. As seen in Fig. 5, all micelles and antimicrobials at the four concentrations had a superior antibiofilm activity compared to the control; however, no clear trend regarding increasing concentrations was seen, nor an improvement of antibiofilm activity of the antimicrobials when conjugated to the micelles. These results could be explained by the micelle's inability to inhibit bacterial adhesion either by cell surface-binding, inhibition of adhesion-related proteins or change of surface hydrophobicity.

**Fig. 6:**
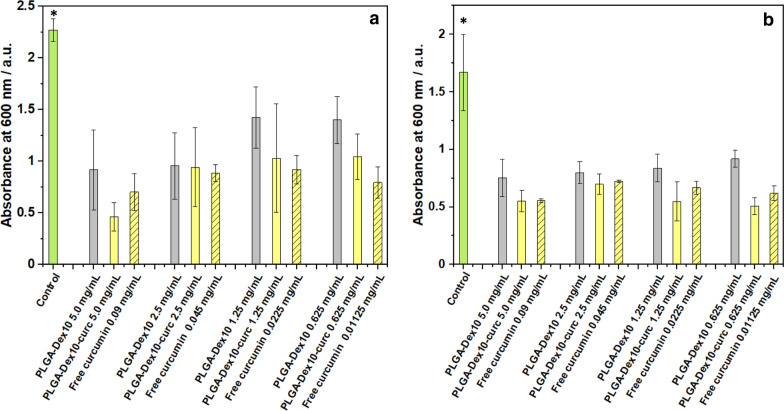
Biofilm biomass quantification using crystal violet staining of pre-formed 24 h of Gram-negative *P.* *fluorescens* (**a**) and Gram-negative *P.* *putida* (**b**) in the presence of micelles, curcumin-functionalised micelles and free curcumin. Control corresponds to the addition of a bacterial culture medium instead of micelles/curcumin solution. Error bars are expressed in terms of standard deviation. Asterisk means statistical significance (p < 0.05)

Another critical aspect of the antibiofilm capacity of any material is the ability to disrupt existent pre-formed biofilms. To this end, biofilms were grown at optimal conditions for 24 h. Then, biofilms were incubated with test micelles and free curcumin, and the biofilm biomass was estimated after 24 h. Lower concentrations of PLGA-Dex_10_-curc significantly disrupted the pre-formed *P.* *fluorescens* and *P.* *putida* biofilms compared to PLGA-Dex_10_ micelles (Fig. 6). A decrease in biomass by 68% was achieved for PLGA-Dex_10_-curc at 0.62 mg mL^−1^ against *P.* *fluorescens*. The disruption of established biofilms by PLGA-Dex_10_-curc could have two main causes. The first is the electrostatic disturbance induced by the micelles positive charge, which would readily interact with the negatively charged EPS components and weaken their overall architecture. Considering that free curcumin had a similar profile to PLGA-Dex_10_-curc while being negatively charged due to its phenolic groups, the second and more likely mechanism is the interaction of curcumin with EPS components directly involved with bacterial adhesion. This has already been demonstrated by Singh et al*.* with in silico studies that show a favourable interaction of curcumin with specific inhibition pockets in modulins and curli proteins of *Escherichia* *coli* biofilms [46].

**Fig. 7:**
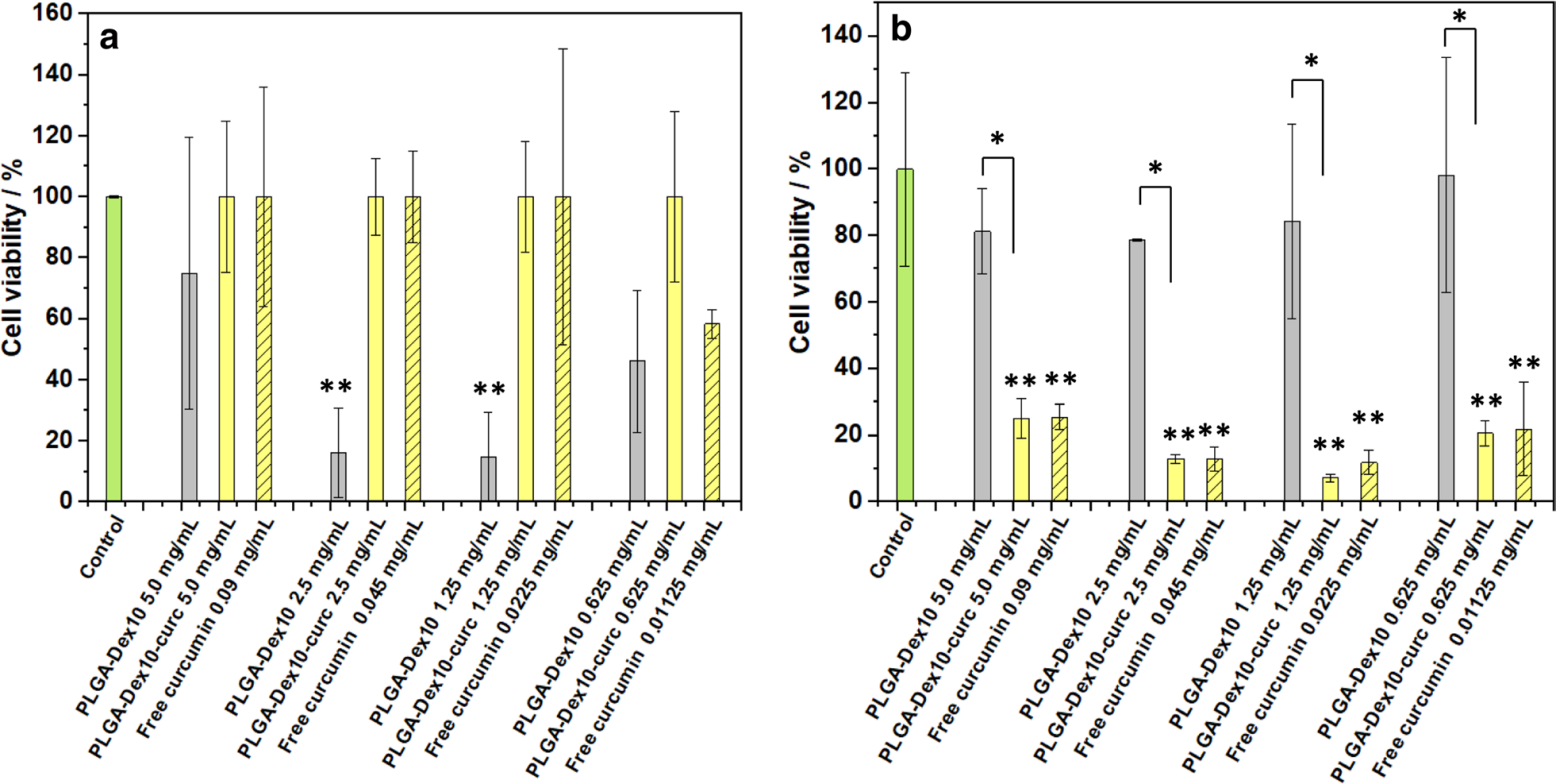
Cell viability assays denoted by absorbance at 495 nm of pre-formed 24 h Gram-negative *P.* *fluorescens* (**a**) and Gram-negative *P.* *putida* (**b**) biofilms in the presence of micelles, curcumin-functionalised micelles and free curcumin. Control corresponds to the addition of a bacterial culture medium instead of micelles/curcumin solution. Error bars are expressed in terms of standard deviation*.* Asterisk means statistical significance (p < 0.05); Double asterisk means statistical significance in comparison with control (p < 0.05)

Whereas biofilm biomass estimation is a good indication of antibiofilm capacity, the integrity of sessile bacterial cells also gives important indications on the mechanism of action of each micelle type. The methylthiazolydiphenyltetrazolium bromide (MTT) assay was used to assess the bacterial cell viability of cells embedded in *P.* *fluorescens* and *P.* *putida* biofilms, and the results are shown in Fig. 7. When comparing the two strains, very distinct patterns are observed; for *P.* *fluorescens* (Fig. 7a), PLGA-Dex_10_ was capable of decreasing cell viability significantly to 14% and 16% at 1.25 mg mL^−1^ and 2.5 mg mL^−1^, respectively. These results confirm this micelle’s unexpected high antibacterial activity also seen for planktonic cells (Fig. 4a). PLGA-Dex-curc did not exhibit antibacterial activity towards sessile cells for *P.* *fluorescens*. Conversely, *P.* *putida* biofilms (Fig. 7b) were not affected at all by PLGA-Dex_10_ micelles, in agreement with the pattern seen for planktonic cells (Fig. 4d). On the other hand, PLGA-Dex_10_-curc micelles reduced cell viability to 21%, 7%, 13% and 25% at 0.62, 1.25, 2.50 and 5.00 mg mL^−1^, respectively. Nevertheless, the functionalised micelle did not outperform free curcumin. The disparity between the trends seen for the crystal violet and MTT assays for the pre-formed biofilms is due to the fact that the former evaluates the overall biomass and the latter unveils whether the embedded bacteria are alive or not. Mild EPS inhibition does not necessarily correlate to the presence of active bacteria, as seen in these experiments.

**Fig. 8:**
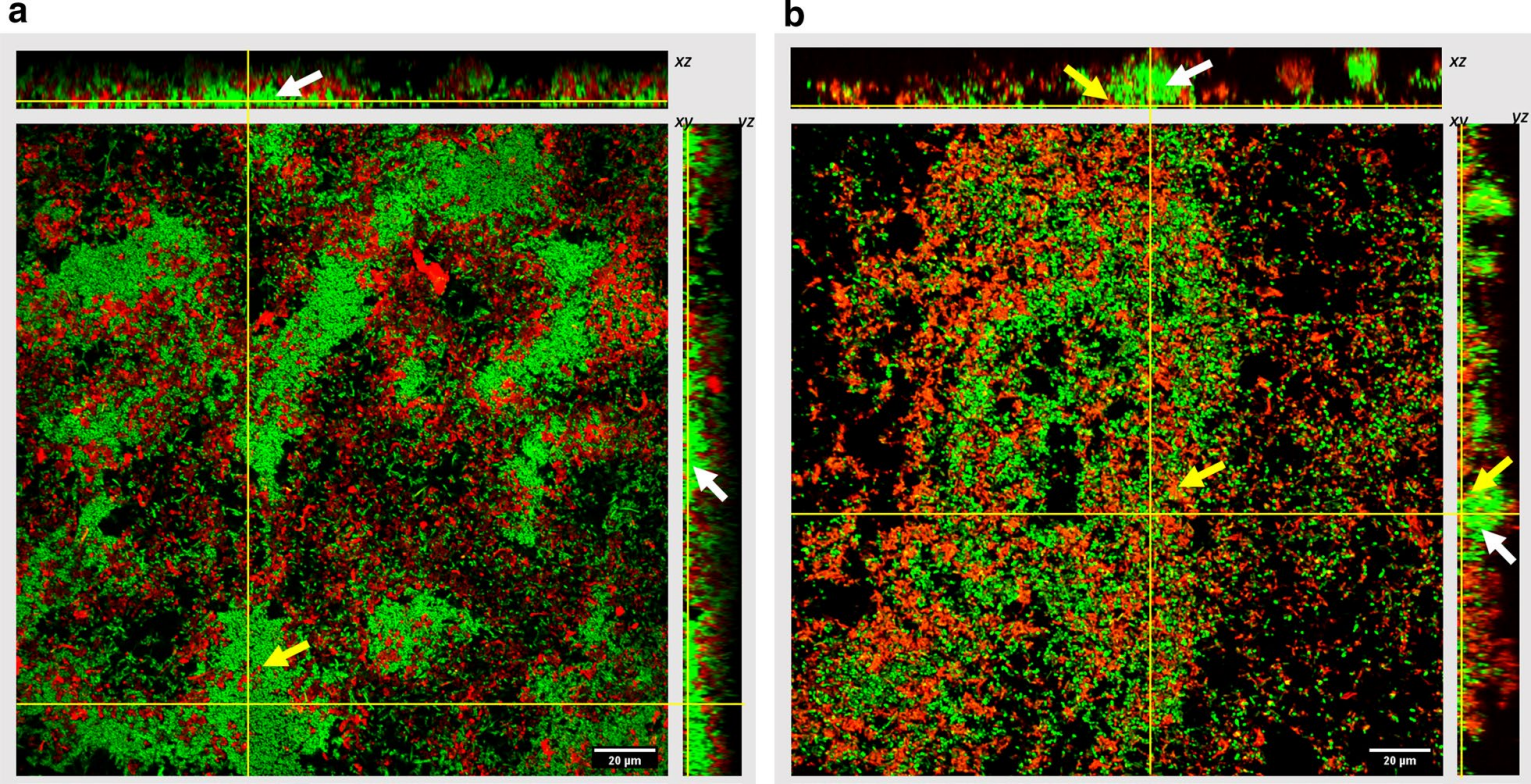
CLSM images of 24 h *P.* *fluorescens* (**a**) and *P.* *putida* (**b**) biofilms (green) after exposure to fluorescently labelled PLGA-Dex_10_ micelles (red). Upper and side panels represent z-stack images of the xz and yz planes, respectively. White arrows indicate microcolonies; yellow arrows indicate micelles which penetrate within these regions

In order to obtain more insights on how the micelles interact with biofilms and penetrate the EPS matrix, PLGA-Dex_10_ was fluorescently labelled with fluorescein (for imaging mCherry-expressing *P.* *fluorescens*) or rhodamine B (for imaging GFP-expressing *P.* *putida*). Biofilms grown in glass coverslips were exposed to the labelled micelles and then imaged using confocal laser scanning microscopy (CLSM) (Fig. 8). It was observed that the micelles were unable to penetrate densely packed regions of the *P.* *fluorescens* biofilm, indicated by the white arrows in Fig. 8a. Very few micelles can be seen inside these microcolonies as shown by the yellow arrow. On the other hand, they could penetrate deeply into the *P.* *putida* biofilm and within the dense microcolonies (Fig. 8b). Nevertheless, both biofilms display a widely spread lateral distribution of polymeric micelles with low levels of aggregation. This could be a consequence of the high compatibility of the dextran shell with the EPS components, which may facilitate the micelles' intake. This effect was demonstrated by Naha et al*.* in a study of dextran-coated nanozymes in which the authors show that the dextran coating induces natural incorporation of the nanoparticle into the EPS matrix and enhances bacterial killing [27]. In their work, several dextran molecules of distinct sizes for coating purposes were studied, and the one that led to the best antibiofilm activity was the 10 kDa one, which is also the one used in the present study. Here, we were unable to obtain images of PLGA-Dex_10_-curc within the biofilms as curcumin has wide excitation and emission spectra, which hinders the correct assignment of fluorescent signals (Additional file 1: Figure S3).

Interestingly, the fact that PLGA-Dex_10_ micelles did not penetrate *P.* *fluorescens* biofilms as effectively as they did for *P.* *putida* contradicts the fact that they are intrinsically antimicrobial (for both planktonic and sessile cells) for *P.* *fluorescens*. This could correlate with the fact that *P.* *fluorescens* usually produces less biomass in the biofilm state, which would mean fewer hurdles for the micelles’ access to bacterial cells despite the difficulty of diffusion into dense microcolonies. Larger amounts of EPS were shown to increase nanoparticle entrapment in *P.* *putida* biofilms, which may lead to more difficult access to the cells [47].

Overall, the results presented here point to the fact that the antibacterial and antibiofilm activities of both PLGA-Dex_10_ and PLGA-Dex_10_-curc follow tendencies that depend on the bacterial phenotype and biofilm development stage. Although PLGA-Dex_10_ has absolutely no effect on the growth rate of planktonic *P.* *putida* nor in bacterial adhesion/EPS production, it does interfere significantly with *P.* *fluorescens* growth. Moreover, it also decreases the bacterial viability of biofilm cells at some concentrations, in spite of not inhibiting biomass production in developing and 24 h biofilms. This observation demonstrates that the dextran itself or the dextran shell in the form of micelle has some type of surface interaction with cells, slowing down the growth rate and/or inducing bacterial killing. Another possibility is that in near proximity of the cells, micellar disassembly is triggered by the local microenvironment and the copolymer induces a chemical imbalance at the cell surface. This effect has been reported before for amphiphilic micelles [48]. On the other hand, after curcumin functionalisation, the results suggest that the micelle acquires intrinsic antibacterial capacity towards both strains. The antibacterial effect seems to arise exclusively from curcumin for *P.* *putida*, as both PLGA-Dex_10_-curc and free curcumin slow down the growth rate considerably as well as reduce the cell viability at the concentrations tested. Still, curcumin incorporated in micelles performs better than when administered freely at the same equivalent concentration. This effect could be due to an increase in the local concentration of curcumin combined with better transport to the biofilm’s innermost regions, which was already performed by our group previously [40]. Additionally, both curcumin and PLGA-Dex_10_-curc seem to also interfere with biofilm biomass formation.

This work introduces curcumin as a part of the copolymer itself instead of encapsulating it in a hydrophobic cavity. In recent years, some papers have reported curcumin delivery in several ways while using polymeric micelles, however not using the approach presented here. Huang et al., for instance, worked with the nanoencapsulation of curcumin in micelles of a poly(ε-caprolactone)-*block-*poly(aspartic acid) copolymer and tested its antibacterial activity against *P.* *aeruginosa* and *Staphylococcus* *aureus* *(S.* *aureus)*. In both cases, at a micellar concentration of 0.5 mg mL^−1^, the cell viability was mildly affected, reaching around 90%. In our work, at a similar concentration (0.62 mg mL^−1^), we also detected mild growth inhibition (Fig. 7) and a decrease in biofilm cell viability to about 20%. This difference could be related to the mode by which curcumin delivery takes place; whereas encapsulation leads to a steady release of curcumin, the incorporation of curcumin in the micelle shell leads to constant and more frequent contact between the antimicrobial and cells [49].

Similarly, a study focused on antimicrobial photodynamic therapy (aPDT) reported the nanoencapsulation of curcumin into cationic and anionic poly(lactic acid)-based micelles. In the absence of light (conditions where aPDT does not take place) and at a micellar concentration of 260 μmol L^−1^, anionic curcumin-encapsulated micelles did not decrease the number of colony-forming units (CFU) of *Streptococcus* *mutans*, methicillin-resistant *Staphylococcus* *aureus* (MRSA) and *Candida* *albicans*. The log_10_(CFU mL^−1^) was decreased by 1.3–3.9 units for cationic micelles, depending on the strain. It is worth mentioning that whereas the encapsulation efficiency of these micelles was very high (about 65%), in our work the curcumin loading is only 1.8% (w/w), which elevates the micellar concentration needed in order to see an effect [50]. The curcumin-functionalised micelle presented here has the additional advantage that the core remains available for the nanoencapsulation of a second bioactive component, which could lead to possibilities for synergism in future applications.

## Conclusion

In this paper, an amphiphilic block copolymer was synthesised using PLGA and dextran and was used for self-assembly into spherical shaped polymeric micelles of around 100 nm. The micelles’ dextran shell was further functionalised with curcumin and their antibacterial and antibiofilm capacities against *P.* *fluorescens* and *P.* *putida* were evaluated*.* It was found that the PLGA-Dex_10_ micelles have an intrinsic antibacterial activity against *P.* *fluorescens* towards both planktonic and sessile cells. Functionalisation with curcumin (PLGA-Dex_10_-curc) resulted in a strong antibacterial activity against both strains heavily dependent on the micelle concentration. The presence of curcumin on the dextran shell enhanced bacterial growth inhibition compared to free curcumin and PLGA-Dex_10_. The curcumin-functionalised micelles were also efficient at disrupting *P.* *fluorescens* biofilms at low concentrations and decreased *P.* *putida* bacterial cell viability at all concentrations. Images of biofilms obtained by CLSM show well-dispersed micelles uniformly distributed laterally throughout the biofilms, with minimal signs of aggregation. This is especially true for *P.* *putida.* The good dispersibility could be due to the high biocompatibility of the dextran shell of PLGA-Dex_10_ with polysaccharides existent within the biofilm EPS, which can easily incorporate the micelle shell into its architecture.

The work presented here is innovative because it brings the possibility of preparing polymeric nanocarriers that show intrinsic antimicrobial and antibiofilm activity. It is recurrent in the scientific literature the use of polymeric nanocarriers that have the sole purpose of delivering an encapsulated biologically active compound. Here, the intent was to show that the polymeric nanocarrier itself may have interesting biological activities which would add up to the overall antimicrobial activity of a nanosystem comprised of a nanocarrier plus antibiotic. Rather than preparing a curcumin-functionalised micelle with enhanced activity compared to curcumin alone, this study focused on incorporating its bioactivity into the micelle structure. Therefore, this work leaves the perspectives of using PLGA-Dex_10_-curc as a nanocapsule for a second bioactive compound, which could in theory further enhance the antibacterial/antibiofilm activities reported here. Additionally, more information needs to be acquired regarding the mechanisms by which PLGA-Dex_10_-curc inhibits cell and biofilm growth and how these differ from free curcumin’s modes of action.

## Materials and methods

### Materials

The following chemicals were purchased from Sigma Aldrich and utilised as supplied: King B Agar, Peptone (vegetable) No. 1, phosphate-buffered saline (PBS), tetracycline hydrochloride, gentamicin sulfate salt, calcium chloride (CaCl_2_), magnesium chloride (MgCl_2_), magnesium sulfate (MgSO_4_), sodium hydroxide (NaOH), acetic acid, dimethylformamide (DMF), dimethyl sulfoxide (DMSO), *N*,*N*′-dicyclohexylcarbodiimide (DCC), dichloromethane (CH_2_Cl_2_), *N*-hydroxysuccinimide (NHS), EDC, HDMA, NaBH_3_CN, Resomer^®^ RG 504 H PLGA 38,000–54,000 kDa (PLGA-COOH), dextran from *Leuconostoc* *mesenteroides* (molecular weight 9000–11,000 kDa), curcumin, glycerol, Mowiol 4-88, rhodamine B isothiocyanate (RITC) and fluorescein isothiocyanate (FITC). Potassium phosphate dibasic was purchased from Honeywell, Fluka™. Cell proliferation kit I (MTT) was purchased from Roche. The water used in all experiments was MilliQ grade water, purified using an Elga Process Water System.

### Copolymer synthesis and micelle self-assembly

For the preparation of the copolymer containing a hydrophobic moiety of PLGA and a hydrophilic moiety of dextran 10 kDa (PLGA-Dex_10_), both polymers had to be activated prior to the conjugation reaction (Fig. 1). The utilised activation procedure was adapted from the one reported by Raza et al*.* [51]. Dextran was activated via reductive amination; to 360 mg of dextran dissolved in 20 mL of DMSO, 376 mg of NaBH_3_CN in 5 mL of DMSO and 41 mg of HDMA in 5 mL of DMSO were added. The mixture was stirred at room temperature for 24 h, and then further 41 mg of HDMA in 5 mL of DMSO were added. After 24 h, the mixture was dialysed against water for 3 days. For the activation of PLGA, 3 mg of DCC in 1.5 mL of CH_2_Cl_2_ and 3 mg of NHS in 1.5 mL of CH_2_Cl_2_ were added to a solution of 150 mg of PLGA-COOH in 15 mL of CH_2_Cl_2_. The reaction mixture was stirred for 6 h at room temperature, and the solvent was evaporated at room temperature overnight. For the preparation of PLGA-Dex_10_, 360 mg of aminated dextran were dissolved in 25 mL of DMSO, and then 169 mg of PLGA activated with NHS were added under inert N_2_ atmosphere. The reaction proceeded under constant stirring at room temperature for 2 days, and the obtained solution was then dialysed against water for 3 days. During the dialysis step, the reaction mixture became increasingly turbid, as a result of the increasing ratio of water/DMSO. As the copolymer is soluble in DMSO and insoluble in water, it spontaneously formed micelles during dialysis.

Fluorescently labelled PLGA-Dex_10_ micelles were also prepared for confocal microscopy experiments. To this end, RITC and FITC were used as fluorescent labels, and a procedure based on the one reported by Skelly et al*.* was used [52]. To 5 mg of PLGA-Dex_10_ micelle in water at pH 10, 10 mg of RITC or FITC were added. After stirring for 3 h at room temperature, the reaction solution was exhaustively dialysed against water.

### Functionalisation of dextran shell with curcumin

For the functionalisation of the micelle's dextran shell with curcumin, the procedure was adapted from the one reported by Rahimnia et al*.* [53]. Firstly, 30 mg of PLGA-Dex_10_ were dissolved in 3 mL of DMSO, and to this solution, 200 mg of EDC in 1 mL DMSO and 200 mg of NHS in 1 mL of DMSO were added. After stirring for 30 min, 36 mg of curcumin dissolved in 3 mL of DMSO were added, and the reaction was allowed to proceed for 24 h at room temperature. The mixture was then dialysed against water for 1 day, and micelles (PLGA-Dex_10_-curc) were self-assembled again as the solvent was changed from DMSO to water. Cycles of washing using centrifugation and removal of supernatant were also performed to guarantee that all adsorbed curcumin molecules had been removed. A scheme of this reaction is shown in Fig. 1d.

### Micelles characterisation

DLS and Zeta potential measurements were performed in a Zetasizer Nano ZS (Malvern Instruments). Micelle samples in 1 mg mL^−1^ aqueous dispersion were analysed in a folded capillary zeta cell. For size measurements, experiments were run in triplicate with 15 runs per measurement. For zeta potential measurements, experiments were done in triplicate with 15 scans each. SEM imaging was carried out using an SEM FEI Quanta 3D FEG Dual Beam SEM, with samples suspended in water deposited on copper tape and dried overnight at room temperature. The size distribution of nanoparticles was determined using the Fiji software [54]. FTIR was performed in a Bruker Vertex 70 spectrophotometer. Samples in powder form were deposited onto NaCl FTIR cards; spectra were taken from 4000 to 400 cm^−1^ with a resolution of 4 cm^−1^ and 64 scans. For estimation of curcumin loading on micelles after dextran functionalisation, UV–Visible spectra of the functionalised micelles were obtained using a plate reader (SpectraMax iD3, Molecular Devices) with a resolution of 5 nm and a spectral window from 230 to 900 nm, using a PLGA-Dex_10_ suspension of the same concentration as a blank. The absorbance of curcumin at 475 nm was used for quantification with the aid of standard curves using standard curcumin solutions. For curcumin solutions (micelles and standard), 100 μL of the sample was mixed with 10 μL of NaOH 2N for complete dissolution in alkaline water. The quantifications of curcumin loading were done in triplicate. Critical micelle concentration values were obtained through conductivity measurements using a Jenway 4510 Conductivity Meter.

### Bacterial growth kinetics

For bacterial cultivation, *P.* *putida* (PCL 1482) and *P.* *fluorescens* (PCL 1701) were streaked from a glycerol stock onto a King B agar plate containing, respectively, the antibiotic tetracycline (40 μg mL^−1^) and gentamycin (10 μg mL^−1^) and incubated at 30 °C for 24 h. A single colony was used to inoculate a sterile conical flask containing 50 mL of King B media supplemented with the appropriate antibiotics. The culture was incubated for 16–18 h at 30 °C, 200 rpm. The optical density at 600 nm (OD_600_) of the *P.* *putida* and *P.* *fluorescens* overnight cultures were adjusted to 0.001 using sterile King B media supplemented with CaCl_2_ (1.5 mmol L^−1^), MgCl_2_ (1.5 mmol L^−1^) and the appropriate antibiotic.

A bacterial suspension of each strain (150 μL) was then added into 96-well plates. Each row (at least 3 wells) was assigned to a different treatment: positive control (further addition of 50 μL of the bacterial mixture), PLGA-Dex_10_ and PLGA-Dex_10_-curc micelles at final concentrations 0.62, 1.25, 2.50 and 5.00 mg mL^−1^, free curcumin at equivalent final concentration values as found in the corresponding micelle (0.011, 0.022, 0.045 and 0.090 mg mL^−1^) As a negative control, all reagents were added in the absence of a bacteria inoculum. Bacterial growth was monitored using absorbance (OD_600_) measurements hourly in a plate reader (SpectraMax iD3, Molecular Devices). During 24 h, the plates were shaken in between the measurements while maintaining a temperature of 30 °C. Because of the initial turbidity caused by micelles, a baseline of the OD_600_ (calculated as an average of the first 8 time points) was subtracted from each data point.

The described biofilms culture procedure was adopted for all the studies performed on the biofilms, namely for bacterial growth kinetics study and for the assessment of biofilm growth inhibition as well as disruption of pre-existing biofilms.

### Biofilm inhibition assay

After 24 h of bacterial growth, the remaining planktonic bacterial suspensions were removed and the biofilms formed were washed twice with water to remove planktonic and loosely attached cells, then 200 μL of crystal violet solution (0.1% w/v) was added, and the plate was left static for 20 min in the dark. The crystal violet solution was then removed, and the stained biofilms were washed with water five times to remove excess unbound dye. Acetic acid (30%) was added (200 μL) to dissolve the stained biofilms and plates were shaken at 125 rpm in the dark for 25 min. Then, the absorbance values at 600 nm were taken using the plate reader. Once again, the wells that were supplemented with gentamycin (40 μg mL^−1^) were used as blanks. This procedure was adapted from O’Toole [55].

### Pre-formed biofilms assay

*P.* *putida* and *P.* *fluorescens* biofilms were cultivated for 24 h as described previously. After removing the media and washing the biofilm with water, 200 μL of suspensions of micelles (0.625, 1.25, 2.5 and 5.0 mg mL^−1^) and free curcumin (0.01125, 0.0225, 0.045 and 0.09 mg mL^−1^) were added to the biofilms. The 96-well plates were shaken for 24 h at 125 rpm and 30 °C and the suspensions were removed from the wells. After washing the biofilms twice with water, crystal violet staining was carried out with the same previously described procedure.

### Cell viability

In order to assess the viability of biofilm bacterial cells, the MTT assay was used. The activity of bacterial reductases can be observed by the reduction of the MTT bromide to formazan, inducing a colour change from yellow to purple-blue. After biofilm growth in 96-well plates as previously described, the bacterial culture was discarded, and biofilms were washed once with PBS; then each well was filled with 175 µL of PBS and 25 µL of MTT solution (pre-warmed to 30 °C). After 1 h incubation, the wells were emptied, and 200 µL of DMSO was added. The plate was shaken at 125 rpm for 15 min to facilitate solubilisation of formazan crystals. Absorbance was read at 550 nm. At least 3 replicates were carried out for each testing condition.

### Confocal microscopy

For biofilm preparation for confocal microscopy analysis, the bacterial overnight culture was adjusted to OD_600_ of 1 and supplemented with CaCl_2_ to a final concentration of 1.5 mmol L^−1^ and 5 mL was added to a sterile 50 mL centrifuge tube containing a glass coverslip (24 mm × 50 mm) and plugged with sterile cotton wool. Tubes were incubated for 24 h at 30 °C at 100 rpm. Each biofilm-coated glass coverslip was carefully removed from the centrifuge tubes and gently rinsed three times in water. The coverslip was then placed horizontally on a sample holder, and 150 μL of the fluorescently labelled micelle (0.5 mg mL^−1^) was added directly to the biofilm, followed by incubation in the dark for 15 min. After incubation, the biofilms were then gently rinsed 3 times in water. Each coverslip was mounted in Mowiol 4-88 (pH 8.5) mounting medium as described previously by our group [4]**.** Horizontal plane z-stack images were acquired with an Olympus FluorView FV1000 CLSM attached to an inverted Olympus IX81 microscope with a 60 ×/1.35 NA UPL SAPO oil immersion objective (Olympus Optical, Tokyo, Japan). At least 3 image stacks, with a z-step of 1 µm, from each of 3 independent experiments were acquired and used for each analysis. All image files were analysed in Fiji image processing software [54].

## Supplementary Information

**Additional file 1**: **Figure S1**. FTIR spectra of dextran, PLGA-COOH and PLGA-Dex_10_ copolymer. Carbonyl stretching band from PLGA-COOH is maintained in PLGA-Dex_10_. Band derived from O-H stretching is also maintained from dextran to PLGA-Dex_10_. Bands in the region between 1600 cm^-1^ to 1000 cm^-1^ in the spectrum of PLGA-COOH had their intensities decreased in the spectrum of PLGA-Dex_10_ possibly due to a concentration effect, as dextran is also abundant in the copolymer.** Figure S2**. FTIR of micelles. Bands derived from curcumin such as C-O bending and C-O stretching (phenol) are conserved in the spectrum of PLGA-Dex_10_-curc, thus confirming the incorporation of the compound in the micelle’s structure. ** Table S1**. Infrared absorption bands assignment of micelles. **Figure S3**. CLSM images of GFP-expressing *P. putida* (A), PLGA-Dex_10_-curc micelles (B), the merged image generated with both *P. putida* and micelles (C) and inset of an area displaying bleedthrough between channels, seen as yellow spots which cannot be assigned correctly to either bacteria or micelles (D).

## Data Availability

The data generated and analysed during the current study is provided in the manuscript and is available from the corresponding authors on reasonable request.

## Abbreviations

AHL: Acyl-homoserine lactone
aPDT: Antimicrobial photodynamic therapy
CFU: Colony-forming units
CLSM: Confocal laser scanning microscopy
CMC: Critical micellar concentration
DCC: *N*,*N*′-Dicyclohexylcarbodiimide
Dex_10_: 10 KDa dextran
DLS: Dynamic light scattering
DMF: Dimethylformamide
DMSO: Dimethyl sulfoxide
EDC: *N*-(3-Dimethylaminopropyl)-*N*′-ethylcarbodiimide hydrochloride
EPS: Extracellular polymeric substances
FITC: Fluorescein isothiocyanate
FTIR: Fourier-transform infrared
HDMA: Hexamethylenediamine
MRSA: Methicillin-resistant *Staphylococcus* *aureus*
MTT: Methylthiazolydiphenyltetrazolium bromide
NHS: *N*-Hydroxysuccinimide
NP: Nanoparticle
OD_600_: Optical density at 600 nm
PBS: Phosphate-buffered saline
PLGA: Poly(lactic-*co*-glycolic acid)
PLGA-COOH: Poly(d, l-lactide-*co*-glycolide) with a carboxylic acid termination
QS: Quorum sensing
RITC: Rhodamine B isothiocyanate
SEM: Scanning laser microscopy
