# Supplementary material for: Synthesis and self-assembly of curcumin-modified amphiphilic polymeric micelles with antibacterial activity
Source: J Nanobiotechnology. 2021 Apr 13;19:104. doi: 10.1186/s12951-021-00851-2 (PMC8045376; doi:10.1186/s12951-021-00851-2)
Supplement: Supplementary file 1 — Additional file 1: Figure S1. FTIR spectra of dextran, PLGA-COOH and PLGA-Dex10 copolymer. Carbonyl stretching band from PLGA-COOH is maintained in PLGA-Dex10. Band derived from O-H stretching is also maintained from dextran to PLGA-Dex10. Bands in the region between 1600 cm-1 to 1000 cm-1 in the spectrum of PLGA-COOH had their intensities decreased in the spectrum of PLGA-Dex10 possibly due to a concentration effect, as dextran is also abundant in the copolymer. Figure S2. FTIR of micelles. Bands derived from curcumin such as C-O bending and C-O stretching (phenol) are conserved in the spectrum of PLGA-Dex10-curc, thus confirming the incorporation of the compound in the micelle’s structure. Table S1. Infrared absorption bands assignment of micelles. Figure S3. CLSM images of GFP-expressing P. putida (A), PLGA-Dex10-curc micelles (B), the merged image generated with both P. putida and micelles (C) and inset of an area displaying bleedthrough between channels, seen as yellow spots which cannot be assigned correctly to either bacteria or micelles (D). [file 12951_2021_851_MOESM1_ESM.docx]

**Additional file 1**

Synthesis and self-assembly of curcumin-modified amphiphilic polymeric micelles with antibacterial activity

Caio H. N. Barros,^a,b^ Dishon W. Hiebner,^a,c^ Stephanie Fulaz,^a^ Stefania Vitale,^a,d^ Laura Quinn,^a^ Eoin Casey^a*^

^a^School of Chemical and Bioprocess Engineering, University College Dublin, Ireland

*Corresponding author

^b^Current address: National Institute for Bioprocessing Research and Training (NIBRT), Dublin, Ireland.

^c^ Current address: School of Pharmacy and Biomolecular Sciences, Irish Centre for Vascular Biology, Royal College of Surgeons in Ireland, Dublin, Ireland

^d^Current address: Université de Strasbourg, CNRS, ISIS, 8 allée Gaspard Monge, 67000 Strasbourg, France


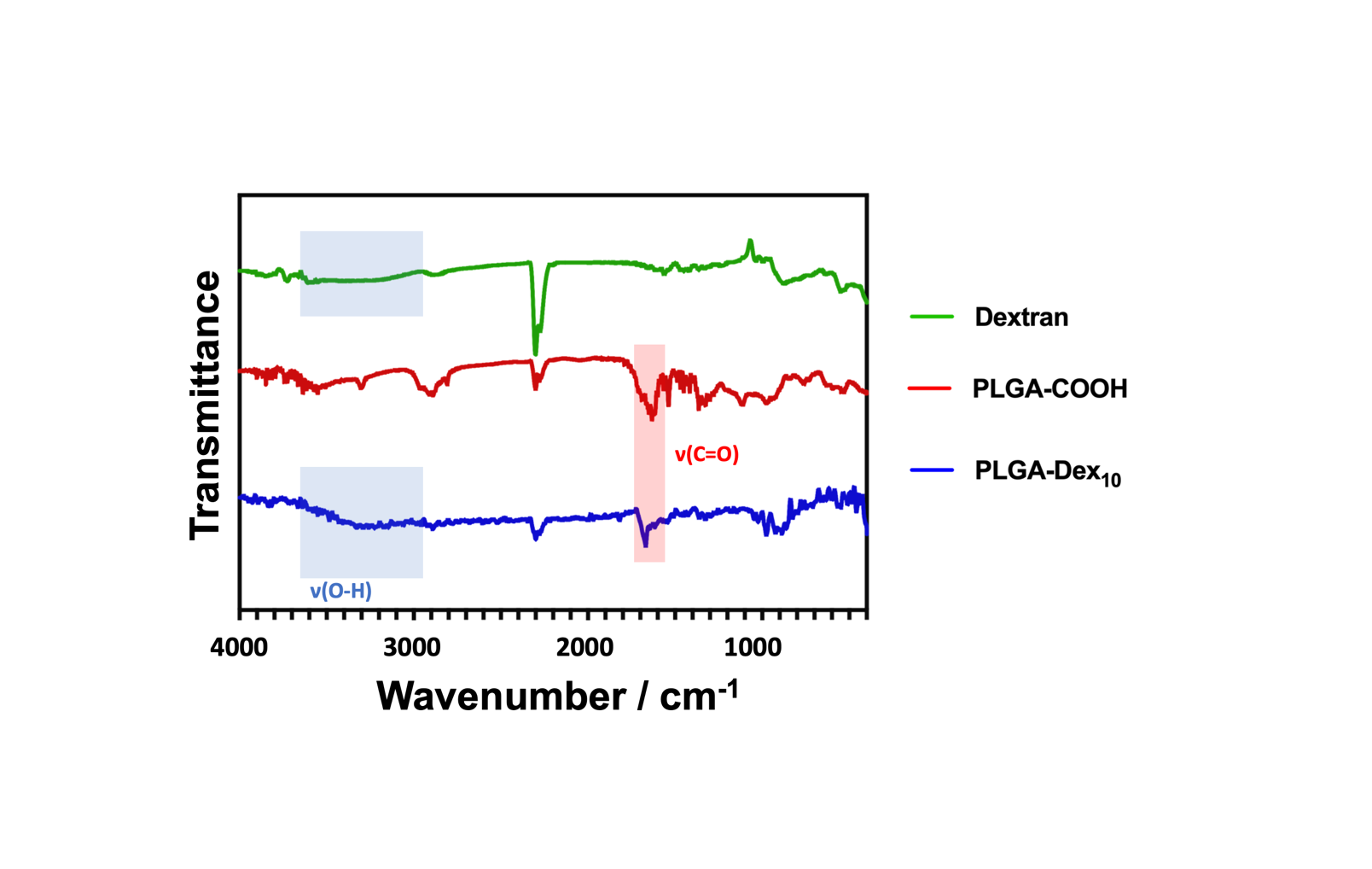


Figure S1. FTIR spectra of dextran, PLGA-COOH and PLGA-Dex_10_ copolymer. Carbonyl stretching band from PLGA-COOH is maintained in PLGA-Dex_10_. Band derived from O-H stretching is also maintained from dextran to PLGA-Dex_10_. Bands in the region between 1600 cm^-1^ to 1000 cm^-1^ in the spectrum of PLGA-COOH had their intensities decreased in the spectrum of PLGA-Dex_10_ possibly due to a concentration effect, as dextran is also abundant in the copolymer.


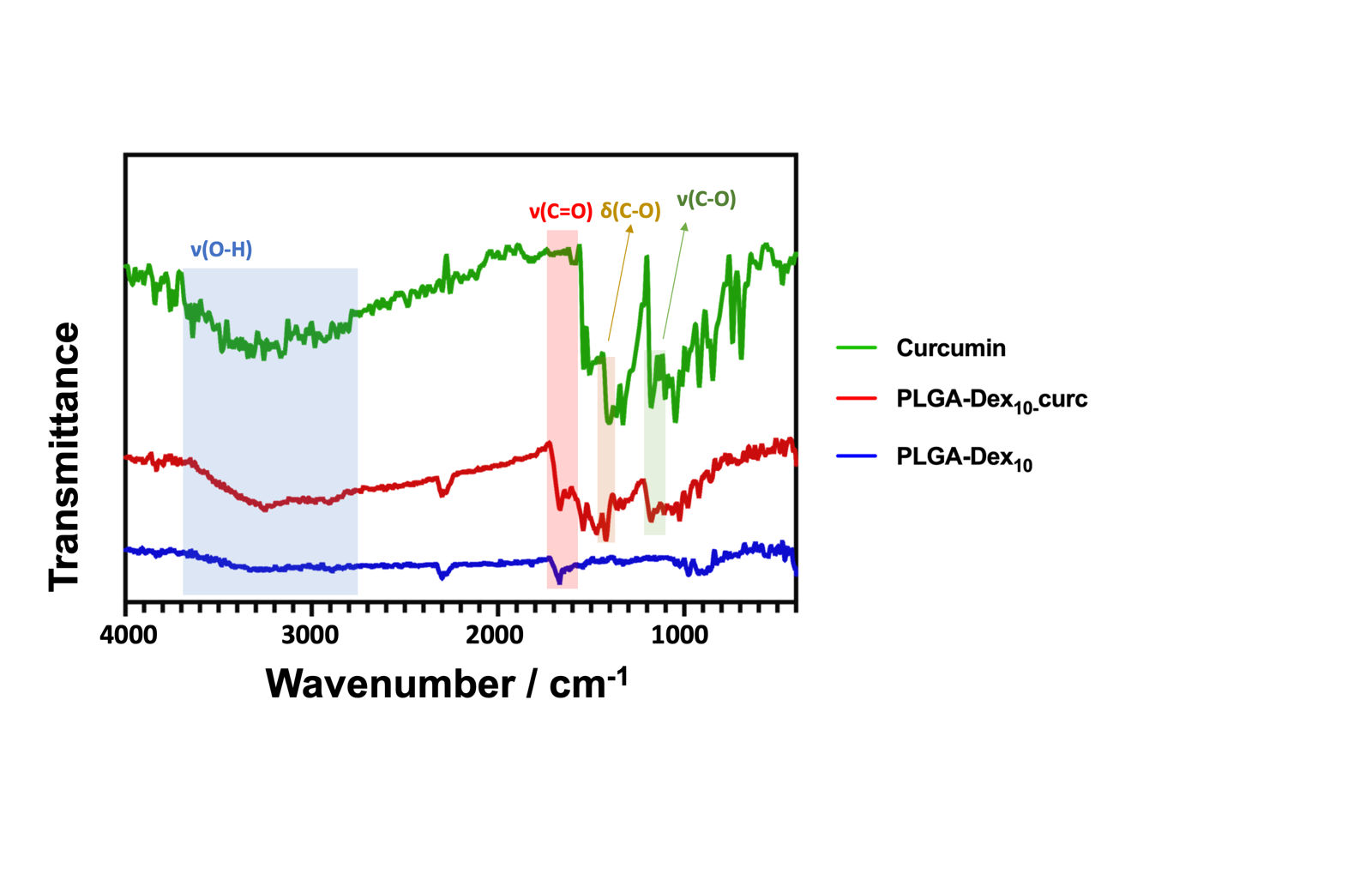


Figure S2. FTIR of micelles. Bands derived from curcumin such as C-O bending and C-O stretching (phenol) are conserved in the spectrum of PLGA-Dex_10_-curc, thus confirming the incorporation of the compound in the micelle’s structure.

Table S1. Infrared absorption bands assignment of micelles

| **Vibrational mode** | **PLGA-Dex_10_** | **PLGA-Dex_10_-curc** | **Curcumin** |
| --- | --- | --- | --- |
| O-H stretching | 3331 cm^-1^ | 3255 cm^-1^ |  |
| C=O stretching | 1670 cm^-1^ | 1668 cm^-1^ | 1610 cm^-1^ |
| C-O stretching (phenol) | --- | 1179 cm^-1^ | 1181 cm^-1^ |
| C=C stretching (alkene) | --- | 1629 cm^-1^ |  |
| C-O bending (enol) |  | 1422 cm^-1^ | 1417 cm^-1^ |
|  |  |  |  |


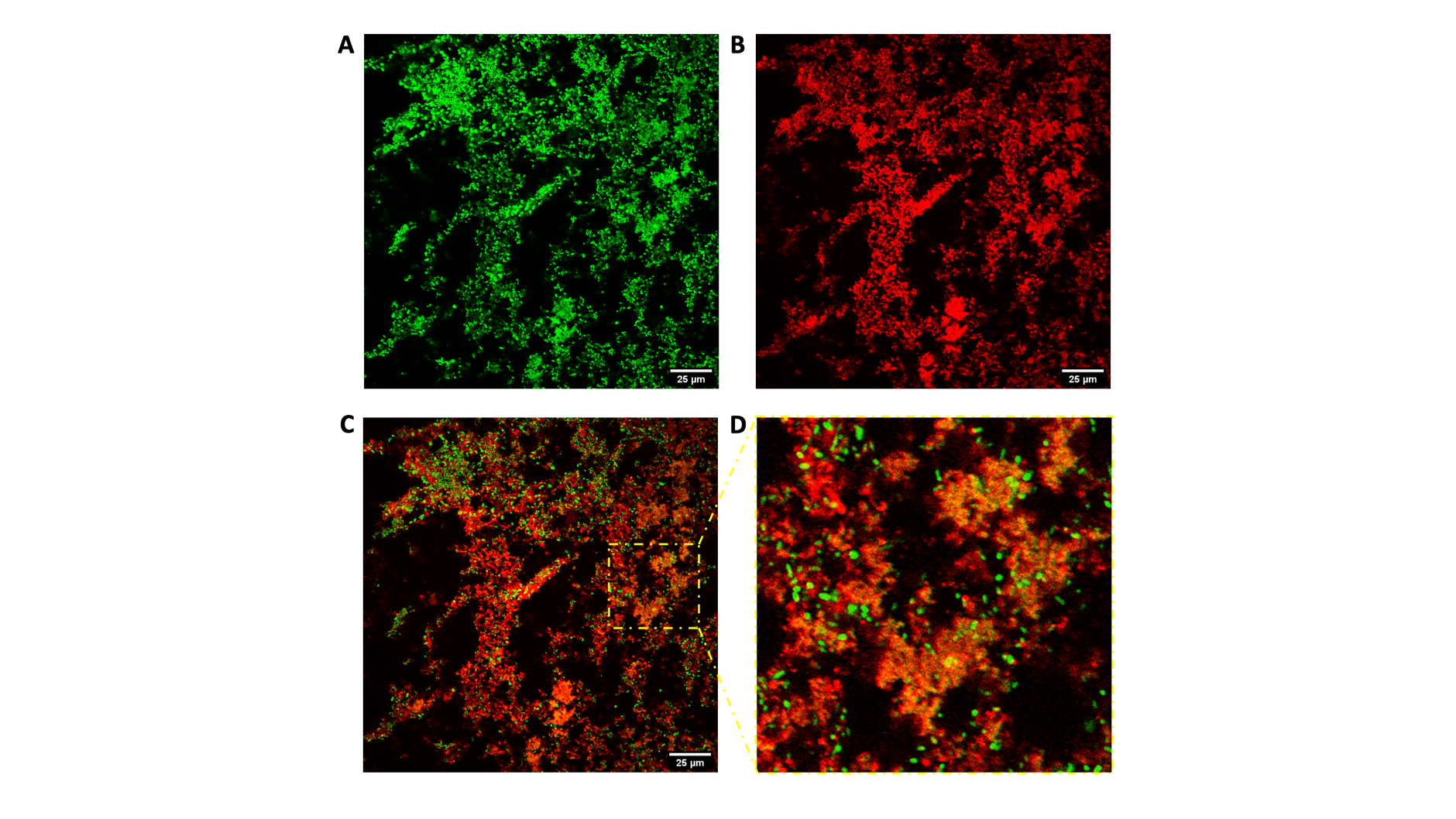


Figure S3. CLSM images of GFP-expressing *P. putida* (A), PLGA-Dex_10_-curc micelles (B), the merged image generated with both *P. putida* and micelles (C) and inset of an area displaying bleedthrough between channels, seen as yellow spots which cannot be assigned correctly to either bacteria or micelles (D).
